# Application of a Saliva‐Based Liquid Biopsy for the Detection of HPV in Patients With Oral Cavity Squamous Cell Carcinoma (OCSCC)

**DOI:** 10.1002/hed.70003

**Published:** 2025-08-05

**Authors:** Michela Bulfoni, Alessandro Tel, Beatrice Krpan, Barbara Marcon, Maria De Martino, Giorgia Vesca, Cinzia Lombardo, Stefania Marzinotto, Emanuele Nencioni, Salvatore Sembronio, Corrado Pipan, Massimo Robiony, Francesco Curcio

**Affiliations:** ^1^ Department of Medicine University of Udine Udine Italy; ^2^ Department of Head and Neck and Neuroscience, Academic Hospital of Udine Clinic of Maxillofacial Surgery Udine Italy; ^3^ Department of Laboratory Medicine Friuli Centrale Healthcare University Hospital (ASUFC) Udine Italy; ^4^ Biofarma Group Srl Mereto di Tomba Udine Italy; ^5^ Public Health Laboratory Friuli Centrale Healthcare University Hospital (ASUFC) Udine Italy

**Keywords:** human papilloma virus (HPV), molecular biology, oral cavity squamous cell carcinoma (OCSCC), saliva, screening test

## Abstract

**Background:**

Human papilloma virus (HPV) is a DNA virus capable of infecting mucous membranes. In most cases, the infection is cleared by the immune system, but a prolonged exposure to HPV can progress to cancer. 40%–60% of head and neck squamous cell carcinomas (HNSCC) are linked to HPV, which is considered a risk factor especially among young people in industrialized countries. HNSCC are not identified early due to their slow growth and their locations not being easy to see. Despite the prognostic value of HPV, the use of HPV‐DNA as a diagnostic marker is not fully developed. HPV‐DNA can be detected in saliva specimens of patients with HPV‐driven cancers. Considering this, we employed saliva samples to optimize a non‐invasive RT‐PCR assay for HPV infection and prove the hypothesis that the presence of HPV DNA represents a risk factor for Oral Cavity Squamous Cell Carcinoma (OCSCC). The potential of this work is to highlight how an HPV screening program based on salivary testing can be useful for early cancer detection and patient monitoring.

**Methods:**

In this retrospective study, 127 patients and 93 control samples were tested for the presence of HPV in self‐collected saliva specimens. Viral DNA was extracted from saliva using an automated instrument. A multiplex RT‐PCR was employed for the detection of the (28 most frequent HPV Types). Patients' demographics were collected in a clinical database. Statistics were performed by STATA16, and significance was set at *p* < 0.05.

**Results:**

Most patients had a diagnosis of OCSCC (49.1%), 38%–9% of which involved the tongue. 9.6% resulted positive for HPV DNA in saliva, specifically for high‐risk subtypes (42.9% type 58; 28.6% types 45, 59, 39 and 9.6% type 16, 18). HPV+patients were compared to those who were found negative. HPV infection was related to the TNM stage, especially with pT2N1 (*p ≤* 0.002) and with primary vs. relapsed tumors (*p =* 0.004). Independent of site, the assay reached a sensitivity of 91.7%, a specificity of 100%, and an agreement of 98.5%, compared to the oropharyngeal swab (*Cohen's Kappa* = 0.947; *n =* 65). Test PPV was 100% (95% CI 71.5–100), while NPV was 98.1% (95% CI 90.1–100).

**Conclusions:**

Our findings indicate that salivary HPV testing is a non‐invasive and convenient test that could become part of routine clinical management for HPV infection of the oral cavity. This test represents an ideal mode of screening of asymptomatic individuals and a long‐term monitoring tool for HPV‐driven cancer patients.

## 1 Introduction

Human papilloma virus (HPV) is a small, non‐enveloped DNA virus capable of infecting the skin or mucous membranes. In most cases, the infection is cleared by the immune system. However, exposure to HPV can lead to persistent infection which, if left untreated, can progress to cancer. Clearance is T‐cell mediated, and individuals with impaired cellular immunity have a higher risk of cancer development.

Head and neck squamous cell carcinomas (HNSCC) are a major public health problem worldwide, with an estimated incidence of 400 000 new cases each year. The etiological role of tobacco and alcohol is well established, just as micronutrient deficiencies and poor oral hygiene are associated with an increased risk of developing these neoplasms [1, 2, 3, 4].

Squamous cell carcinomas of the oral cavity (OCSCC) and oropharynx (OPSCC) associated with human papillomavirus (HPV) infection are now a well‐characterized entity, which predominantly affects males, young or middle‐aged, non‐smokers [3, 5, 6, 7]. The increase in incidence mainly affects the younger cohorts, with greater male gender variability, and is due to the increase in infection rates by high‐risk HPV strains (16 and 18), secondary to changes in sexual habits. Recent studies in a sample of American adults demonstrated a prevalence of approximately 10% for any genotype and 6.5% for high‐risk genotypes [8, 9, 10] According to some estimates, the proportion of HPV‐related OPSCC in high‐income countries is between 65% and 83% [9, 10, 11, 12].

In contrast to cervical cancer, there is no gold‐standard method for identifying high‐risk HPV infections in head and neck cancers. In addition, the clinical specificity of the finding of high‐risk HPV is difficult to assess because the reported manifestation of HPV infection in high‐grade head and neck epithelial lesions is highly variable [13]. However, the most common method for HPV identification consists of brushing the lesion with a swab, followed by PCR molecular characterization of the E6 and E7 regions of the viral genome [14, 15, 16].

When expressed, the HPV oncogenes E6 and E7 induce the degradation of the tumor suppressors p53 and pRB, which leads to uncontrolled cellular proliferation. E6 and E7 interact with the mechanisms of apoptosis and regulation of the host cell cycle. These two genes are often used in molecular approaches for PCR detection of HPV [14, 17].

HPV is not the only risk factor for head and neck cancer. To better stratify patients for the risk of neoplasia, the study of the oral microbiome is important, since alterations of the same associated with the long‐term use of tobacco products (such as the overgrowth of some bacterial species, such as *Porphyromonas, Prevotella* and *Fusobacterium*) are involved in various phases of tumor growth, influencing both the onset and invasiveness, as well as the response to antineoplastic therapy [18, 19, 20].

Although the HPV vaccination campaign is expected to modify the natural history of the disease, this will (probably happen in the coming decades). Therefore, several authors have advocated the start of screening campaigns aimed at identifying the presence of HPV in the oral cavity of people with high risk [15]. To effectively conduct this screening campaign, it is necessary to employ tools that are able to identify subjects at risk in the least invasive way possible, but with high sensitivity. To date, screening for these malignancies has been performed by general practitioners or dentists through visual inspection and physical examination to identify early lesions [6, 21, 22].

HPV DNA can be detected in saliva samples when cells are actively producing viral particles or when infected cells die and release the virus back into the saliva. The possibility of obtaining an early diagnosis thanks to the identification of the viral infection in the epithelial cells exfoliated from the oral cavity or oropharynx present in the saliva is supported by an ever‐increasing number of scientific evidence [15, 16, 23, 24, 25]. Given the need to screen the general population and to develop a rapid, inexpensive, and minimally invasive procedure to diagnose HPV infection of the oral cavity, the objective of this study is to identify and determine the frequency of the virus HPV in saliva samples from oral squamous cell carcinoma patients using a PCR‐based molecular procedure, CE‐IVD. The search for HPV will be carried out using molecular biology tests which allow the presence of high and low oncogenic risk HPV DNA to be detected. In case of positivity, typing will be performed to identify the 28 strains responsible for the infection. Considering all these aspects, we employed a saliva based real‐time PCR assay to test the hypothesis that the presence of HPV DNA represents a risk factor for head and neck squamous cell carcinoma.

Moreover, despite the hot topic, no systematic studies are available concerning saliva collection and analysis validation based on both patient and asymptomatic or healthy groups.

## 2 Materials and Methods

### 2.1 Study Design and Participants

In this retrospective study, 127 patients' and 93 control samples were tested for the presence of HPV in self‐collected saliva specimens (age ≥ 18). All patients had a diagnosis of OCSCC and were followed clinically in the surgical oncology outpatient care of the Oral and Maxillofacial Surgery Department of the Academic Hospital of Udine. The study was approved by the local Institutional Review Board (*prot*. *IRB 124/2023; Tit III cl 13 fasc 5/2023*). All samples collected were anonymized using an alpha‐numeric identification code.

In a subgroup of 60 samples, the comparison between HPV detection results obtained on oropharyngeal swab (OP), which is considered the gold standard, and on salivary samples was performed.

For patients, we also looked for any correlation between HPV positivity, HPV type, and clinical‐demographical data.

### 2.2 Oro‐Pharyngeal (OP) and Saliva Sample Collection

OP specimens were collected by mid‐turbinate swabbing of the oral cavity. A flocked swab (*ESwab Copan; Copan Diagnostics Inc. Murrieta, California*) was used for the collection of all OP clinical samples. For saliva collection, a non‐invasive method was executed by making a self‐collected specimen in a tube container. The conserving solution contained in the saliva collection tubes was provided by Biofarma Srl (*Mereto di Tomba, Udine, Italy*) and it is protected by intellectual property (*Italian Patent Application No. 102021000017297, filed on July 1, 2021 by Biofarma Srl*). The specific saline composition and the pH value made this solution optimal for the stabilization and conservation of nucleic acids in saliva. All samples tested were stored at −80°C until the time of the DNA extraction.

### 2.3 Viral DNA Extraction

Viral DNA was extracted and purified starting both from OP specimens and saliva samples using an automated extractor (*SeeGene Inc. Irvine, California*), according to the manufacturer's instructions.

Briefly, DNA was extracted using the NIMBUS IVD automated platform in combination with the STARMag 96 X 4 Universal Cartridge Kit (Seegene, Korea), which employs magnetic bead‐based technology for nucleic acid purification. A total of 300 μL of sample was processed per reaction, and DNA was eluted in a final volume of 100 μL. The system automates all steps of the extraction workflow, including cell lysis, nucleic acid binding, washing, and elution, ensuring high reproducibility and minimal hands‐on time. Magnetic beads selectively bind nucleic acids under optimized buffer conditions, enabling efficient removal of inhibitors and contaminants and yielding high‐quality DNA suitable for downstream molecular applications.

DNA extracted was eluted into a multiwall plate and immediately employed for the multiplex PCR.

### 2.4 Multiplex Real Time PCR Assay

A multiplex real time PCR (RT‐PCR) was employed for the detection of the 28 most frequent HPV types, using the 28 *Allplex II* detection kit, following the company's instructions (*SeeGene Inc. Irvine, California*).

The Allplex HPV28 Detection assay is a multiplex real‐time PCR system designed to simultaneously detect 19 high‐risk and 9 low‐risk HPV genotypes. The assay targets specific sequences using genotype‐specific primers and probes distributed across two separate master mixes, each covering a distinct panel of genotypes. PCR reactions were carried out on a CFX96 Real‐Time PCR Detection System (Bio‐Rad) in a total volume of 20 μL, including 5 μL of purified nucleic acid and 15 μL of PCR Mastermix. The assay includes two internal controls: one to monitor the amplification process within each sample, and one included in the reaction mix to verify the correct performance of both positive and negative control reactions (Figure S1).

The thermal profile consisted of 45 amplification cycles, with fluorescence acquisition at 60°C, 72°C, and 83°C. Data were analyzed using Seegene Viewer software, which applies a proprietary algorithm for genotype identification. Melting curve analysis was used to discriminate HPV types based on genotype‐specific melting temperatures (Tm), allowing for precise differentiation of single and multiple infections within a single reaction well.

### 2.5 Statistics

Kappa coefficient was presented to estimate agreement between OP swab and saliva test results, with its 95% CI. Descriptive statistics for categorical variables are presented as number (percent) and for continuous variables as mean ± standard deviation (SD) or median (interquartile range; IQR). Normality was assessed using the Shapiro–Wilk test. Comparisons between categorical variables were performed using the Chi‐square or Exact Fisher test, as appropriate. For continuous variable comparisons between two groups were done using the *t*‐test or Mann–Whitney *U* test, comparisons among groups were done using ANOVA or Kruskal‐Wallis test, as appropriate. For patients, Cox regression was used to estimate association between test positivity and HPV type, after the assumptions were verified. All analyses were performed by STATA 16 statistical software, and statistical significance was set at *p* < 0.05. HPV prevalence was calculated as the proportion of HPV‐positive cases (as determined by salivary assay) over the total number of patients tested.

## 3 Results

### 3.1 Population and Diagnostic Performances of the Tests

In this study, a total of 220 saliva samples of patients with a diagnosis of OCSCC were tested for the molecular detection of HPV.

The overall prevalence of HPV infection determined on saliva specimens was 9.6% (21/220) (95% CI 6.0–14.2).

The clinical and pathological factors of patients are shown in Table 1. Compared to females, a significantly higher proportion of males were observed to have HPV DNA present in their saliva (13.7% vs. 4.9%; *p* = 0.026), the vast majority of which were a high‐risk type (79%). The most common subtype isolated was HPV‐58, which was present in 42.9% of all patients tested. All other subtypes occurred in a small minority of patients.

**TABLE 1:** Demographic and clinical characteristics of subjects.

*N* = 220	
Patients, *n* (%)	127 (57.7)
Controls, *n* (%)	93 (42.3)
Age, mean ± SD	65.7 ± 13.7
Female sex, *n* (%)	103 (46.8)
Squamous cancer, *n* (%)	108 (49.1)
Tongue	42 (38.9)
Maxilla	14 (13.0)
Mandible	10 (9.3)
Floor of the mouth	9 (8.3)
Retromolar trigone	9 (8.3)
Lower lip	6 (5.6)
Buccal mucosa	5 (4.6)
Salivary ducts	4 (3.7)
Palate	2 (1.9)
Oral cavity	1 (0.9)
Cheek	1 (0.9)
Upper lip	1 (0.9)
Gingival mucosa	1 (0.9)
Mandibular buccal mucosa	1 (0.9)
Parotid gland:	1 (0.9)
Tonsil	1 (0.9)
TNM (Tumor‐Nodes‐Metastasis) classification, *n*/*N* (%)	
pT1N0	24/86 (27.9)
pT1N0	12/86 (13.9)
pT2N0	9/86 (10.5)
pT4aN1	8/86 (9.3)
pT2N1	6/86 (7.0)
pT3N3b	5/86 (5.8)
pT1Nx	3/86 (3.5)
pT3N0	3/86 (3.5)
Tis	3/86 (3.5)
pT1N1	2/86 (2.3)
pT3N1	2/86 (2.3)
pT4aN2b	2/86 (2.3)
pT2N3b	1/86 (1.2)
pT2(m)rpN0	1/86 (1.2)
rpT2N1	1/86 (1.2)
pT2Nx	1/86 (1.2)
rpT1N0	1/86 (1.2)
rpT2	1/86 (1.2)
rpT1	1/86 (1.2)
Node positive disease, *n*/*N* (%)	26/86 (30.2)
Primary tumor, *n*/*N* (%)	100/107 (93.5)
Recurrence, *n*/*N* (%)	7/107 (6.5)
HPV in saliva, *n* (%)	21 (9.6)
Type, *n*/*N* (%)	
16	1 (4.8)
18	1 (4.8)
35, 6	1 (4.8)
45, 59, 39	6 (28.6)
53	1 (4.8)
58	9 (42.9)
6	1 (4.8)
68	1 (4.8)
Risk classification, *n*/*N* (%)	
High	15/15 (100)

**FIGURE 1:**
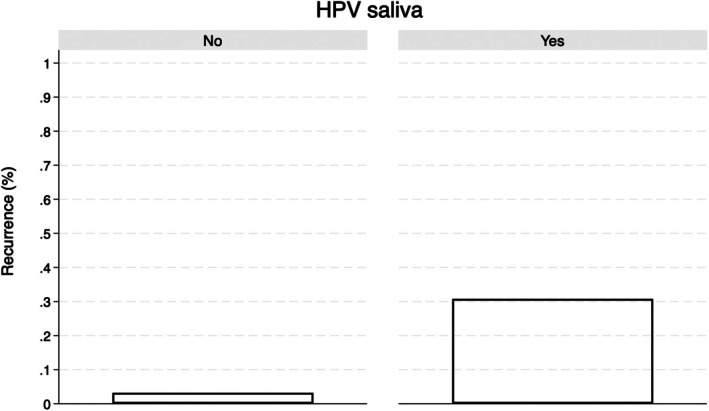
Bar graph showing the recurrence rate (%) based on the presence of HPV in saliva. The “Yes” group exhibits a significantly higher recurrence rate compared to the “No” group, which shows minimal recurrence.

Most patients had a diagnosis of OCSCC (49.1%), 38%–9% of which involving the tongue. 9.6% resulted positive for HPV DNA in saliva, specifically for high‐risk subtypes (42.9% type 58; 28.6% types 45, 59, 39 and 9.6% type 16, 18). HPV+patients were compared to those were found negative. HPV infection was related to the TNM stage, especially with pT2N1 (*p* ≤ 0.002) and with primary vs. relapsed tumors (*p* = 0.004; Figure 1).

### 3.2 Validation of the Analytical Procedure: Performance Metrics of Salivary Test vs. OP Swab

Analyses of concordance were conducted comparing results obtained from saliva and OP swab in 65 samples, collected from the same subject. Among these 65 patients, 75.4% were affected by squamous cell carcinoma.

**FIGURE 2:**
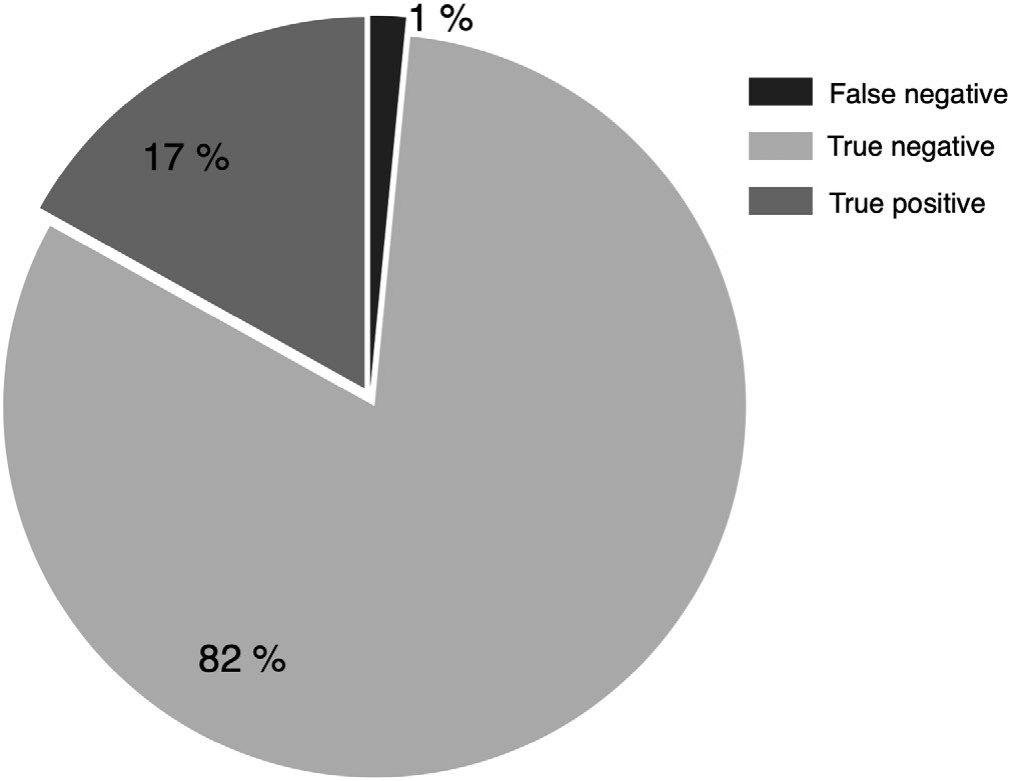
Pie chart illustrating the distribution of diagnostic test results. The majority (82%) represent true negative results, while 17% correspond to true positive results. A small fraction (1%) are false negatives.

The agreement between the two tests was 98.46% with a Cohen's Kappa coefficient of 0.947 (95% CI 0.845–1.000). The test sensitivity was 91.7% (95% CI 61.5–99.8) and the specificity was 100% (95% CI 93.3–100) (Figure 2). The test showed a Positive predicted value (PPV) of 100% (95% CI 71.5–100) and a Negative predicted value (NPV) of 98.1% (95% CI 90.1–100).

## 4 Discussion

In this study we demonstrated the use of a saliva‐based assay for detecting HPV DNA in patients with OCSCC. The collection method proposed was extremely easy to use, and as the collection solution is stable at room temperature for long periods of time, it allowed for batching of samples for future qPCR testing, as demonstrated in our retrospective study.

The prevalence of HPV infection in our study population was 9.6%, which is considerably higher than a recent meta‐analysis in which a prevalence of 32% was documented among patients with HNSCC [1, 7, 8, 12]. A saliva‐based assay could detect HPV DNA present anywhere in the oral cavity or oropharynx, in contrast with a tissue biopsy, which would only detect HPV DNA in selected cells [26, 27, 28, 29].

The relationship between HPV and HNSCC, especially the oropharynx, has been elucidated in several studies [15, 23, 27, 30, 31]. Indeed, most of the knowledge is related to studies considering oropharyngeal cancer (OPSCC). Several pathogenic mechanisms have been described to understand the biology of HPV in relation to the development of neoplasms of the oro‐pharyngeal tract. Other than the well‐known actions of oncogenic proteins E6 and E7, HPV was proven to exert multiple effects on the tumor microenvironment (TME), including an immunosuppressive effect for the reduced expression of MHC‐1 and an increase of immune‐inhibitory agents such as IL‐10 and TGF‐ß [32]. Other studied mechanisms include improved cell survival due to the nuclear localization of caspase 8 and BAK degradation, immune system evasion for the increased expression of PD‐1/PD‐L1, CTLA‐4, genomic instability (APOBEC mutational signature has been described), and establishing replicative immortality through MYC and hTERT activation [30].

HPV‐positive HNSCC exhibit distinctive cytogenic features which include a difference in the translocations formed, suggesting a substantially higher genomic instability compared with HPV‐negative HNSCC cells, yet both cell types demonstrate an increase in aneuploidy [31].

Compared with oropharynx, studies suggest that HPV‐positive oral cavity squamocellular carcinomas (OCSCC) cases are infrequent and less impactful [5], yet our study demonstrates that several non‐conventional HPV genotypes are associated with oral cancers. In 2023, a metanalysis was conducted to assess the overall HPV+OCSCC prevalence, including more than 5000 patients. Results indicated that the HPV+OCSCC proportion had a substantial variation from 0% to 37%, with an overall prevalence of 6%, with the tongue being the predominant site for HPV location [1, 10, 12], considering that the posterior tongue includes the lingual tonsil with a tissue characterization similar to the oropharynx. Other studies point out that the most commonly associated genotypes for HPV infection in the oral cavity are HPV16, 18, 26, 31, 33, 35, 45, 56, 58, 59, and 67 [9]. However, it may be considered that the proportion of HPV‐positive OCSCC is underestimated due to a variety of reasons: while the detection of HPV E6/E7 mRNA expression is considered the golden standard to diagnose HPV infection by some authors, this technique is expensive and technically difficult, as it requires fresh frozen material, which is not always available, and is not compatible with the usual processing of the intraoperative sampling, fixed using formaline [23, 32]. Thus, the most established and practical essay is measuring cytoplasmic and nuclear immunoreactivity for p16, which is known to be a surrogate marker for the incidence of HPV in OPSCC, yet the method has only been used for OPSCC and has not been validated for OCSCC [3, 29]. Additionally, p16 may have different roles in the pathogenesis of OPSCC and OCSCC. Thus, other studies suggested combining p16 overexpression and HPV‐DNA PCR to increase HPV detection sensibility and specificity [13, 17, 26].

In contrast with available literature, preliminary results of our study show that the prevalence of HPV infection in patients with OCSCC might be substantially higher, being 9.6% in our sample. In addition, care should be taken to examine genotypic variants which are not typically associated with HNSCC, including 35, 36, 45, 59, 39, 53, 58, 68; thus, a wide genotype panel should be implemented to search for less conventional variants. Moreover, given the high concordance between OP swab and liquid biopsy PCR, this method should be routinely proposed to patients with a diagnosis of carcinoma before surgery, when the tissue release of virions in saliva is maximal, owing to its simplicity of collection and ease of processing, as the tubes used to collect saliva can preserve intact the viral genome for several days.

In fact, to prevent degradation and ensure high‐quality DNA for downstream analyses, all samples in this study were collected directly into a nucleic acid preservation solution. This stabilizing medium rapidly inhibits enzymatic activity while also fluidifying the saliva, improving its compatibility with automated extraction protocols. Following extraction, DNA samples were either processed immediately for PCR or stored at −80°C to preserve their integrity over time. The validity and robustness of this collection strategy had already been established in our laboratory during the COVID‐19 pandemic, where it yielded highly reliable results across a range of molecular applications [33].

Considering the method validation, the agreement between the two tests was 98.46% with a Cohen's Kappa coefficient of 0.947 (95% CI 0.845–1.000). The test sensitivity was 91.7% (95% CI 61.5–99.8) and the specificity was 100% (95% CI 93.3–100). The test showed both a positive predicted value (PPV) of 100% (95% CI 71.5–100) and a negative predicted value (NPV) of 98.1% (95% CI 90.1–100).

Additionally, from this preliminary data, a correlation between the TNM stage and the presence of infection seems not to be present, as HPV positive samples occurred both in early and advanced stages, yet this conclusion might be biased by the relatively low number of HPV positive OCSCC samples.

The prevalence of HPV in our study was also higher in men and in patients with tumors arising from the tongue. Likewise, in our study, patients who tested positive for HPV were more likely to present with lymph node positive disease.

Interestingly, considering the high concordance between HPV‐DNA PCR extracted from saliva, a positive salivary HPV assay could support the surgeon in exploring the oral cavity and could potentially act as an additional surrogate marker when a tissue biopsy is unavailable. Furthermore, a saliva‐based assay could become a post‐treatment surveillance tool to monitor the risk of recurrence in OPSCC, considering that the overexpression of p16 has demonstrated poor performance as a prognostic marker for overall survival in OCSCC, and it has been un‐recommended to use it as a tool for OCSCC in study trials.

As a result of its sensitivity and its high specificity, as well as for the ease of collection and processing, a salivary liquid biopsy with HPV characterization could be useful in the preoperative evaluation of patients affected by OCSCC. Further studies are needed to define a potential role for salivary HPV‐DNA as a marker of disease recurrence for HPV‐positive OCSCC.

## 5 Conclusions

Molecular methods based on saliva samples must be implemented in the clinical monitoring of both patients at risk and those who have already developed a pre‐neoplastic lesion. Starting from these considerations, our study lays the foundation for the development of HPV molecular biology assays able to identify the population at risk through screening campaigns.

## Conflicts of Interest

The authors declare no conflicts of interest.

## Supporting information


**Figure S1:** Representative amplification and melting temperature (Tm) curves from the multiplex real‐time PCR assay for the detection and genotyping of Human Papillomavirus (HPV) (*Allplex HPV28 Detection kit, SeeGene*). Panels (A–C) show fluorescence amplification curves: (A) single HPV 18 infection; (B) co‐infection with multiple HPV genotypes, each detected via genotype‐specific fluorophores; (C) HPV‐negative sample showing amplification of the internal controls only (one for each reaction mix). Panels (D–F) display the corresponding Tm curves for genotype discrimination: (D) distinct peak matching HPV 18; (E) multiple peaks consistent with the mixed infection (e.g., HPV 16, 53, 58, 82); (F) internal controls' peaks only. This assay enables both qualitative detection and precise genotyping of HPV in a single reaction.

## Data Availability

The data that support the findings of this study are available from the corresponding author upon reasonable request.
