# Supplementary material for: Application of a Saliva‐Based Liquid Biopsy for the Detection of HPV in Patients With Oral Cavity Squamous Cell Carcinoma (OCSCC)
Source: Head Neck. 2025 Aug 5;48(1):56–63. doi: 10.1002/hed.70003 (PMC12703555; doi:10.1002/hed.70003)
Supplement: Supplementary file 1 — Figure S1: Representative amplification and melting temperature (Tm) curves from the multiplex real‐time PCR assay for the detection and genotyping of Human Papillomavirus (HPV) (Allplex HPV28 Detection kit, SeeGene). Panels (A–C) show fluorescence amplification curves: (A) single HPV 18 infection; (B) co‐infection with multiple HPV genotypes, each detected via genotype‐specific fluorophores; (C) HPV‐negative sample showing amplification of the internal controls only (one for each reaction mix). Panels (D–F) display the corresponding Tm curves for genotype discrimination: (D) distinct peak matching HPV 18; (E) multiple peaks consistent with the mixed infection (e.g., HPV 16, 53, 58, 82); (F) internal controls' peaks only. This assay enables both qualitative detection and precise genotyping of HPV in a single reaction. [file HED-48-56-s001.docx]

**Supplementary Figure 1**


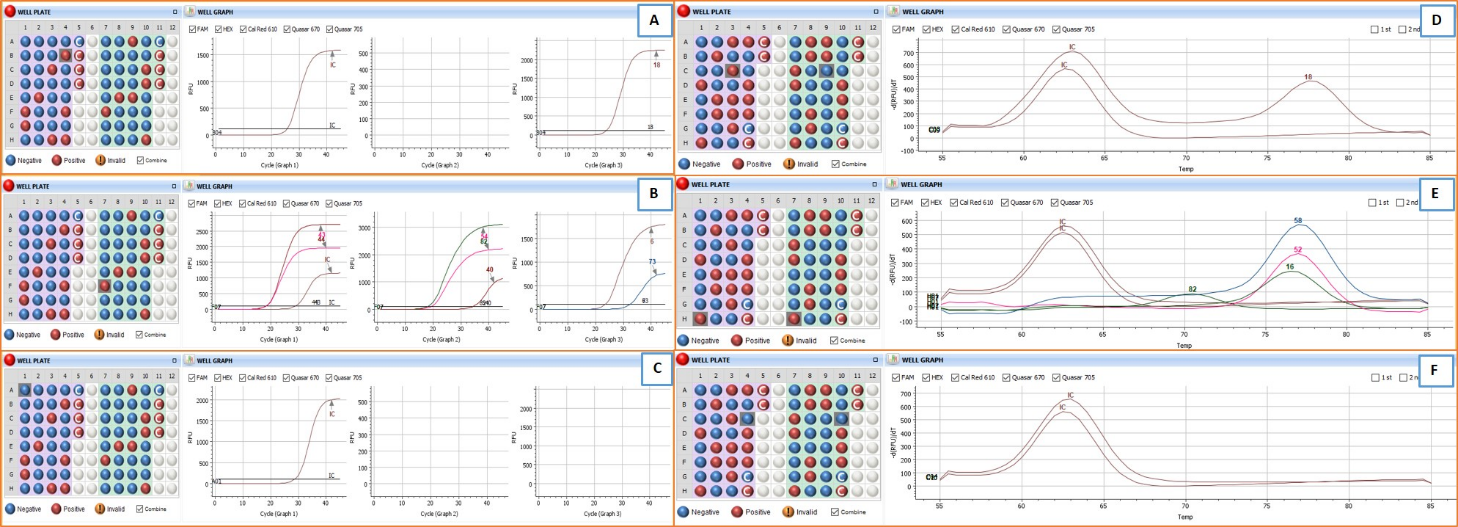


Representative amplification and melting temperature (Tm) curves from the multiplex real-time PCR assay for the detection and genotyping of Human Papillomavirus (HPV) (*Allplex HPV28 Detection kit, SeeGene*). Panels A–C show fluorescence amplification curves: (A) single HPV 18 infection; (B) co-infection with multiple HPV genotypes, each detected via genotype-specific fluorophores; (C) HPV-negative sample showing amplification of the internal controls only (one for each reaction mix). Panels D–F display the corresponding Tm curves for genotype discrimination: (D) distinct peak matching HPV 18; (E) multiple peaks consistent with the mixed infection (e.g., HPV 16, 53, 58, 82); (F) internal controls’ peaks only. This assay enables both qualitative detection and precise genotyping of HPV in a single reaction.
